# Psychotropic Drug Use Before And During Physical Restraint For Mechanically Ventilated Critically Ill Adults

**DOI:** 10.1186/2197-425X-3-S1-A28

**Published:** 2015-10-01

**Authors:** L Burry, M Guenette, T Farquharson, A Cheung, S Mehta, M Traille, I Mantas, L Rose

**Affiliations:** Mount Sinai Hospital, Pharmacy, Toronto, Canada; Mount Sinai Hospital, Medicine, Toronto, Canada; Mount Sinai Hospital, Nursing, Toronto, Canada; University of Toronto, Nursing, Toronto, Canada

## Background

Physical restraint (PR) is common in Canadian intensive care units (ICUs); used in up to 76% of mechanically ventilated patients.[1] Psychotropic drugs may minimize PR used for agitation, promoting patient safety and preventing treatment interference. No data describes the psychotropic drug use around the time of PR application.

## Objective

To characterize alterations to psychotropic drug regimens 1 hour prior and 6 hours after PR application for critically ill, mechanically ventilated adults.

## Methods

Prospective single centre observational study (Feb 2014 to Jan 2015). Eligible patients were physically restrained while receiving mechanical ventilation. Psychotropic drug data were collected 1 hour before PR and for 6 hours after PR. We recorded PR indications, psychotropic drug interventions including new drug initiation, dose increase, dose decrease, and drug cessation. We documented total duration of PR, Sedation Agitation Scale (SAS) scores and presence of delirium (Intensive Care Delirium Screening Checklist score ≥4). We used McNemar's tests to compare across time points.

## Results

We enrolled 93 patients meeting our inclusion criteria. Mean age 59 years, 53% male, and 65% medical admissions. Median (IQR) mechanical ventilation duration was 5 (2, 9) days. 111 indications for PR included prevention of treatment interference (90, 81%), agitation (13, 12%), physical violence towards staff (5, 5%), and self-harm (3, 3%). Median (IQR) duration of restraint application was 21 (9, 70) hours. of the 69 patients with delirium screening documented, 23 (33%) were delirious. More patients received a psychotropic drug intervention after PR (52 vs 80, P < 0.001) and more patients received an opioid after PR (19 vs 50, P < 0.001). Use of other drugs did not differ (Table 1). Sedation profile of the 51 patients with SAS scores documented pre and post is shown in Table 2. Adverse events during PR were uncommon with unintentional device removal in 7 (8%) patients.

**Table 1:** Table 1

Drug therapy [n (%)] (N = 93)	pre-PR	post-PR	P
Any psychotropic drug intervention	52 (56)	80 (86)	< 0.01
a) New drug/increase b)Drug stop/decrease	a) 42 (45) b) 10 (11)	a) 69 (74) b) 11 (12)	a) < 0.01 b) 1.00
No change	23 (25)	7 (8)	< 0.01
No psychotropic drug	18 (19)	6 (6)	< 0.01
Drug class (received >1 drug)			
Opioids	19 (20)	50 (54)	< 0.01
Non-benzodiazepine sedatives	22 (24)	27 (29)	0.44
Benzodiazepines	20 (22)	29 (31)	0.18
Antipsychotics	4 (4)	10 (11)	0.11

**Table 2:** Table 2

Sedation (N = 51)	n (%)
Appropriate pre/post (SAS 3-4)	12 (24)
Achieved SAS 3-4 post	8 (16)
Agitated pre/post (SAS 5-7)	8 (16)
Oversedated pre/post (SAS 1-2)	8 (16)
SAS 3-4 pre: SAS 5-7 post	6 (12)
SAS 5-7 pre: SAS 1-2 post	5 (10)
SAS 1-2 pre: SAS 5-7 post	4 (8)

## Conclusions

Psychotropic drug interventions were more common after PR application with opioids used most frequently suggesting priority of analgesia over sedation. Most patients had a SAS score indicating appropriate or oversedation in the hour prior suggesting PR was used more often as a preemptive approach.
